# Compliance with Regulatory Standard 32 in the units of a general hospital

**DOI:** 10.47626/1679-4435-2020-704

**Published:** 2023-02-03

**Authors:** Mariana Santos Romão, Pamela Franco Silva, Angelica Augusta Grigoli-Dominato

**Affiliations:** 1 Faculdade de Medicina de Presidente Prudente, Unoeste, Presidente Prudente, SP, Brazil.; 2 Biomedicina, Faculdade de Ciências da Saúde, Universidade do Oeste Paulista (Unoeste), Presidente Prudente, SP, Brazil.

**Keywords:** occupational diseases, occupational health, protective devices, accidents, occupational, legislation, doenças profissionais, saúde do trabalhador, equipamentos de proteção, acidentes de trabalho, legislação

## Abstract

**Introduction:**

In Brazil, the Ministry of Labor and Employment created Regulatory Standard number 32 (Norma Regulamentadora 32, NR-32), instituted by Ordinance 485, of November 11^th^, 2005. It establishes measures to protect workers’ safety and health in any health service.

**Objectives:**

To quantify employees’ compliance with NR-32 in the several units of a general hospital in the inland of the state of São Paulo, Brazil, reducing accidents related to work activity and allowing for the establishment of its fulfillment.

**Methods:**

This is an exploratory study with a qualitative and quantitative approach of data. Semi-structured questionnaires were applied to the volunteers.

**Results:**

Thirty-eight volunteers participated, who were divided into a group of professionals with a higher education degree (53.5%), consisting of nurses, physicians, and resident students, and another group of professionals with technical and high school degree and nursing assistants. Among the volunteers, 96.4% reported that they knew NR-32 and 39.2% reported having experienced an occupational accident in the period before the study. The use of personal protective equipment was reported by 88% of volunteers, and needle recapping by 7.1% of them.

**Conclusions:**

Assimilation of NR-32 by health care professionals, regardless of schooling, as well as its application within the hospital, may be a way of protection against occupational accidents during the development of work activities. Allied to this, protection may be extended with constant training of these workers.

## Abstract (pt)

**Introdução:**

No Brasil, o Ministério do Trabalho e Emprego criou a Norma Regulamentadora número 32 (NR-32), instituída pela Portaria 485, de 11 de novembro de 2005. Ela estabelece as medidas para proteger a segurança e a saúde dos trabalhadores em qualquer serviço de saúde.

**Objetivos:**

Quantificar a adesão dos funcionários à NR-32 nos diversos setores de um hospital geral no interior do estado de São Paulo, reduzindo os acidentes relacionados à atividade laboral e permitindo o estabelecimento do cumprimento da norma.

**Métodos:**

Estudo exploratório de abordagem qualitativa e quantitativa dos dados. Foram aplicados questionários semiestruturados aos voluntários.

**Resultados:**

Totalizaram 28 voluntários, divididos em um grupo de profissionais atuantes com curso superior (53,5%), composto por enfermeiros, médicos e estudantes de residências, e em outro grupo de profissionais com nível de ensino médio e técnico e auxiliares de enfermagem. Entre os voluntários, 96,4% disseram conhecer a NR-32, e ocorreram 39,2% acidentes de trabalho no período anterior ao estudo. O uso de equipamento de proteção individual foi relatado por 88% dos voluntários, e o reencape de agulhas, por 7,1%.

**Conclusões:**

A assimilação da NR-32 entre os profissionais da área da saúde, independentemente da escolaridade, assim como sua aplicação dentro do hospital, parece ser uma maneira de proteção contra acidentes de trabalho durante o desenvolvimento das atividades laborais. Aliado a isso, a proteção pode ser estendida com a constante capacitação desses trabalhadores.

## INTRODUCTION

Occupational health has been focus of attention since the late 17th century, when disease symptoms started to be associated with occupational exposures. The first treatise on work-related diseases, entitled *De Morbis Artificum Diatriba*, was written in 1700 by Bernardino Ramazzini, who is considered the father of occupational medicine. The book describes the interrelationship between professional activity and disease, establishing the specific treatment and preventive measures.^1^ This work was translated into Portuguese (by Dr. Raimundo Estrêla) and made it possible to identify some occupations and health problems presented by workers. Thus, it called attention to the need for physicians to know previous and current occupations of their patients in order to diagnose and treat properly.^2^

In the early 18th century, period of the Industrial Revolution in England, new equipment was developed and the number of occupational accidents increased significantly. At that time, when labor conditions led to health damages, there was the emergence of occupational medicine.^3^

According to the Secretariat of Labor Inspection of the Brazilian Ministry of Labor and Employment, in modern times, with the different activities developed by the several occupational segments, there are still many cases of accidents and resulting diseases. Therefore, prevention helps protect against accidents and exposures during occupational practice, preventing the development of occupational diseases. This prevention should be performed in stages, including worker’s knowledge on the risks arising from their professional activity, measures to minimize their exposure to these risks and practices,^4^ as well as frequent training, which lead to knowledge on work procedures that are essential to prevent incidents and their recurrence.^5^

However, the epidemiological importance of occupational accidents has increased and, in Brazil, they are considered extremely severe. These events usually accounts for approximately 25% of injuries from external causes treated in emergency services.^6^ In order to obtain statistical data, all occupational accidents started to be notified by the System of Information for Notifable Conditions through Ordinance n. 777 of April 28^th^, 2004, which provides on technical procedures for mandatory notification of injuries to worker’s health in a specific sentinel service network of the Brazilian Unified Health System (Sistema Único de Saúde, SUS).^7^ It is worth highlighting that mandatory notification is extremely important, since it provides health agencies and systems with information on involvement of their health care providers and may take promotion, protection and control measures related to these accidents, in addition to develop new care protocols to workers.

Therefore, the Brazilian Ministry of Labor and Employment created Regulatory Standard 32 (Norma Reguladora, NR-32) instituted by Ordinance n. 485, of November 11^th^, 2005, which establishes measures to protect workers’ safety and health in all health services. The responsibility for complying with NR-32 regulations is shared by contractor and contracting parties.^8^ Thus, workers are covered by a specific legislation, related to their protection, since, before that, there was no regulation to support them in case of exposure to risk agents.^9^

There are other ordinances that support health care workers, such as Ordinance n. 1,679, of September 19^th^, 2002, which provides on the structuring of the National Network for Workers’ Comprehensive Health Care of SUS, considering the principles of equity, integrality, and universality and promoting prevention, promotion and recovery of the health of urban and rural workers.^10^ Another resolution in this same framework is Ordinance n. 2,728, of November 11^th^, 2009, which provides on the National Network for Workers’ Comprehensive Health Care, establishing protocols, lines of care, and other instruments that favor integrality in health.^11^ Finally, Ordinance n. 1,823, of August 23^rd^, 2012, instituted the National Workers’ Health Policy, which strengthens occupational health surveillance and articulates technical teams and Workers’ Health Referral Centers (Centros de Referência em Saúde do Trabalhador, CEREST) whenever needed, in order to provide specialized technical assistance, considering their role on matrix support to the entire SUS network.^12^

Therefore, it is possible to observe the joint effort of these competent agencies through strategies and implementation of measures to ensure comprehensive health care to workers. The establishment of legislations and standards is the most adopted method, with NR-32 being one of the main standards established with the purpose of preventing accidents and diseases afecting workers during the practice of work activities. NR-32 recommends adopting preventive measures in each risk situation, as well as training these workers for a safe and efficient work, and should be complied by health services to minimize the worrying rates of occupational accidents.^13^

Hence, with the purpose of warning and reassuring its importance, the literature on the subject was surveyed. In view of the magnitude of NR-32 for health care professionals, the research collected and got acquainted with data on compliance with NR-32 by health care professionals working at a hospital in the city of Presidente Prudente, state of São Paulo, Brazil.

The rationale for reflecting on this theme is allowing for the assessment of the methodology of the activities performed in the hospital in search for a safe work. In any procedure performed by health care professionals, safety is a key factor to prevent occupational accidents. It is possible to re-evoke and reorganize actions according to NR-32 in order to obtain beter results.

The objective of this study was to quantify employees’ compliance with NR-32 in order to reduce accidents related to work activities, enabling to promote its fulfillment, in the several sectors of a general hospital in the inland of the state of São Paulo, Brazil.

## METHODS

### TYPE OF RESEARCH

The research was qualitative, quantitative, and descriptive.

### DETERMINATION OF SPACE AND POPULATION

The study was conducted in a referral general hospital located in the inland of the state of São Paulo, which covers 45 municipalities in Western region of this state and, indirectly, municipalities in the states of Paraná and Mato Grosso do Sul. Currently, the hospital is managed by a Health Social Organization (Organização Social de Saúde, OSS).

The aforementioned hospital has a health system organized with actions based on SUS policy, which are developed by a significant number of health care and administrative professionals.

The collection of data on NR-32 compliance was performed with professionals who work in different hospital units. The application of the two questionnaires to assess employees’ compliance with the standard took place during 6 months, in 2018.

### PROCEDURE

The instruments used were two semi-structured questionnaires developed by the authors, the first of which included general questions for all volunteers, and the other included specific questions for some hospital units (diagnostic imaging and oncology) on NR-32. Interviews were performed concomitantly with the development of work activities, in order to enable a more extensive investigation about volunteers’ reality.

### ETHICAL AND LEGAL ASPECTS

The research was approved by the Research Ethics Commitee of Universidade do Oeste Paulista (Unoeste), pursuant to Resolution n. 466/2012 of the National Health Council and Hospital Ethics Commitee (Certificate of Presentation for Ethical Consideration [Certificado de Apresentação de Apreciação Ética] n. 73758817.6.0000.5515). Participants were invited in their workplace and were included as volunteers afer reading and signing the informed consent form (ICF).

### ANALYSIS OF RESULTS

Results were evaluated by descriptive statistical analysis in tables and relative numbers.

## RESULTS

Initially, 79 professionals were invited; however, 51 refused to participate, and 28 volunteers remained in the study. The sample consisted mainly of women (n = 16, 57.1%) and professionals with higher education (n = 15, 53.5%), followed by those with a technical degree (n = 10, 35.7%), and a high school degree (n = 3, 10.8%). The reported professions were nurses, physicians, residents, nursing assistants, and other functions related to the nursing technician position. Of the volunteers, 21 (75%) reported working for less than 10 years in the aforementioned hospital.

Afer research participants returned the questionnaire, there was a talk about awareness on NR-32 and its importance for workers’ health, as well as on the positive impact that it may have within the workplace.

Volunteers worked in the following hospital units: wards (n = 11, 39.3%), diagnostic imaging (n = 5, 17.9%), oncology (n = 4, 14.3%), emergency department (n = 4, 14.3%), hemodialysis (n = 2, 7.1%), blood bank (n = 1, 3.6%), and obstetric center (n = 1, 3.6%).

Table 1 presents the distribution of the hospital units analyzed in the study stratified by volunteers’ schooling.

**Table 1:** Volunteers’ schooling (%) distributed according to the unit where participants performed their work activity

Unit	Higher education	Technical	High school
Wards	28.6	10.7	-
Oncology	7.1	7.1	-
Emergency department	3.6	-	10.7
Blood bank	3.6	-	-
Hemodialysis	-	7.1	-
Diagnostic imaging	-	10.7	7.1
Obstetric center	3.6	-	-

Of the themes addressed in the general questionnaire, it was observed (Table 2) that 100% of volunteers stated that they knew the risks inherent to their profession. Nearly 86% answered that they were informed on these risks when they were hired. All of them reported having a periodical vaccination program, as well as initial and continuing professional training. However, only 64.4% of respondents answered correctly about proper waste disposal.

**Table 2:** Relative numbers (%) obtained in the general questionnaire about knowledge on Regulatory Standard 32 (NR-32)

Questions	Yes	No	Did not answer	Sometimes	Do not know	Not applicable
Vaccination program	85.7	7.1	3.6	-	3.6	-
Updated vaccination	100.0	-	-	-	-	-
Matched the columns on waste disposal correctly	64.4	17.8	-	-	10.7	7.1
Initial and continuing training program	75.0	10.7	3.6	-	10.7	-
Equipment maintenance	82.2	7.1	-	-	10.7	-
Risks	100.0	-	-	-	-	-
Informed about risks	85.7	14.3	-	-	-	-
Floor in good conditions	85.7	14.3	-	-	-	-

Table 3 shows volunteers’ knowledge about NR-32 and its peculiarities. It was observed that nearly of 20% of volunteers did not answer to questions on definition of the standard, did not describe facilitating factors, and did not address lack of compliance or appropriate procedures in case of an occupational accident. During the study period, no occupational accident was reported. However, nearly 40% of participants reported having experienced this type of accident during the development of work activities since they were hired. The non-occurrence of occupational accidents is related to initial and periodical employees’ training, and nearly 78% reported undergoing standard and regular training. Moreover, the use of personal protective equipment (PPE), which is associated with a decrease in the number of occupational accidents, was higher than 88%, although it was mandatory for the development of safe activities, as well as its immediate replacement as needed. Another way of eliminating accidents for those who manipulate needles is the procedure of not recapping them; however, nearly 7% of participants reported performing this procedure.

**Table 3:** Relative numbers (%) obtained from the general questionnaire on Regulatory Standard 32 (Norma Reguladora 32, NR-32) actions

Questions	Yes	No	Did not answer	Sometimes	Did not answer the open question
Knows NR-32	96.4	3.6	-	-	22.2
Experienced an occupational accident	39.2	60.8	-	-	-
Standard training	89.2	7.1	3.6	-	-
Regular training	67.8	17.8	14.4	-	-
Involves the entire team	82.2	3.6	14.2	-	-
Use of PPE	89.2	10.8	-	-	-
Use of PPE during the last shift	88.0	8.0	4.0	-	-
Maintenance of PPE	84.0	8.0	8.0	-	-
Easily accessible PPE	88.0	4.0	8.0	-	-
Immediate replacement of PPE	76.0	20.0	4.0	-	-
Recaps needles	7.1	85.7	-	7.2	-
Takes of the coat after work shift	78.6	3.6	3.6	14.2	-

PPE = personal protective equipment.

The questionnaires applied in the diagnostic imaging units showed that four (80%) employees are aware that pregnant women should be moved away from work activities and know the radiological protection plan. The individual use of a dosimeter was reported by two (40%) volunteers, and one (20%) reported being informed about radiological risks and protection procedures. The question on monitoring of surfaces afer work shif was not answered in three (60%) questionnaires. Questions on entrance door signaling, presence of a qualified professional, doors properly closed during the procedure were answered positively by all participants.

Volunteers working in the oncology unit answered a questionnaire specific for the unit, and three (75%) reported that pregnant and lactating women are not moved away from work activities. However, four stated (100%) that there was a room specific for preparation of antineoplastic drugs; moreover, participants reported to be aware of the existence of a private restroom (n = 3, 75%), controlled access (n = 4, 100%), external signaling (n = 4, 100%), shielded partition next to patient’s bed (n = 1, 25%).

## DISCUSSION

According to the study, nursing technicians and nurses were more familiar with NR-32, as also reported by Santos Júnior et al.^14^ Female sex also stands out, in line with another study regarding NR-32, which observed the predominance of women in courses on occupational safety and health with training for NR-32.^1^

There is a gap in knowledge about NR-32 among workers at units, implying in risks for individual and collective protection during the development of professional activities in the workplace, not only regarding the use of PPE and collective protection equipment (CPE), but also regarding safety measures. The use of PPE is important to protect employees and patients during health care delivery; thus, it is important to replace and change PPE whenever necessary.

The number of accidents resulting from needle recapping was not reported, but this practice favors accidents, as observed by Marziale et al.,^15^ whose study assessed implantation of NR-32 and control of occupational accident and revealed that the highest percentage of occupational accidents involves needles.

The NR-32 provides several actions aimed at preserving health when performing work activities in the different hospital units.^8^ The diagnostic imaging and oncology units have specific characteristics with regard to risk exposure. Therefore, it is important for professionals to be aware of individual care measures and care measures with patients so that to prevent occupational accidents or contaminations to occur in the long term.

Another important tool addressed by the standard is related to waste disposal. One of atributions of health care professionals is to be familiar with the types of waste, as well as with their separation and final destination, being considered basic knowledge for health care professionals, regardless of schooling. Thus, this needs to be understood by all employees working in the health sector. Inadequate waste disposal may harm both the environment and health care workers, because contamination with biological material or any other type of medical waste may lead to serious consequences for workers’ health.

Although there are still many aspects of NR-32 and its application to be assimilated by professionals working at the hospital under study, the research brought positive results with regard to knowledge and adherence to the standard. The impact of the study on NR-32 increased openness to discussion on this mater, having a positive action on the hospital units. Furthermore, it reinforces the need for constant training by health care professionals so that to prevent gaps in content, learning, and applicability of the themes approached, since updates are inherent to good professional performance.

## CONCLUSIONS

Assimilation of NR-32 by health care professionals, regardless schooling, as well as its application within the hospital, seems to protect against occupational accidents during the development of work activities. Allied to this, protection may be extended with constant training of these workers.

## References

[1] Watanabe E, Razaboni AM, Takayanagui AMM, Machado AA, Castro SM (2015). Avaliação do curso de saúde e segurança do
trabalho – capacitação segundo a NR32. Rev Cult Ext USP.

[2] Ramazzini B (2016). As doenças dos trabalhadores.

[3] Franco-Benatti DM (2011). Acidentes e doenças relacionadas ao trabalho na indústria de
calçados de Franca – SP.

[4] Chagas AMR, Salim CA, Servo LMS (2011). Saúde e segurança no trabalho no Brasil: aspectos institucionais,
sistemas de informação e indicadores.

[5] Organización Internacional del Trabajo (OIT) (2015). Investigación de accidentes del trabajo y enfermedades profesionales:
guía práctica para inspectores del trabajo.

[6] Cavalcante CAA, Cossi MS, Costa RRO, Medeiros SM, Menezes RMP (2015). Análise crítica dos acidentes de trabalho no
brasil. Rev Atençao Saude.

[7] Soares MKP, Fernandes SLSA, Barros VRP (2015). Aplicabilidade da Norma Regulamentadora 32 por profissionais da
saúde no controle de acidentes biológicos: revisão
integrativa. Rev Educ Univ Fed Vale São Francisco.

[8] Confederação Nacional dos Trabalhadores na Saúde (2013). NR-32: boas condições de trabalho exigem saúde e
segurança para o trabalhador(a).

[9] Queiroz C (2006). As implicações da NR-32 no setor de saúde: aspectos
trabalhistas, previdenciários e tributários [Internet].

[10] Brasil, Ministério da Saúde (2002). Portaria GM nº 1679, de 19 de setembro de 2002.

[11] Brasil, Ministério da Saúde (2009). Portaria nº 2.728, de 11 de novembro de 2009.

[12] Brasil, Ministério da Saúde (2012). Portaria nº 1823, de 23 de agosto de 2012 [citado em 25 jun. 2017].

[13] Oliveira JE, Lage KR, Avelar SA (2011). Equipe de enfermagem e os riscos biológicos: Norma Regulamentadora
32 (NR – 32). Rev Enferm Integr.

[14] Santos Jr AG, Santos FR, Furlan MCR, Araújo JC, Arantes MB, Barbosa TS (2015). Norma Regulamentadora 32 no Brasil: revisão integrativa de
literatura. Rev Enferm Cent O Min.

[15] Marziale MHP, Galon T, Cassiolato FL, Girão FB (2011). Implantação da Norma Regulamentadora 32 e o controle dos
acidentes de trabalho. Acta Paul Enferm.

